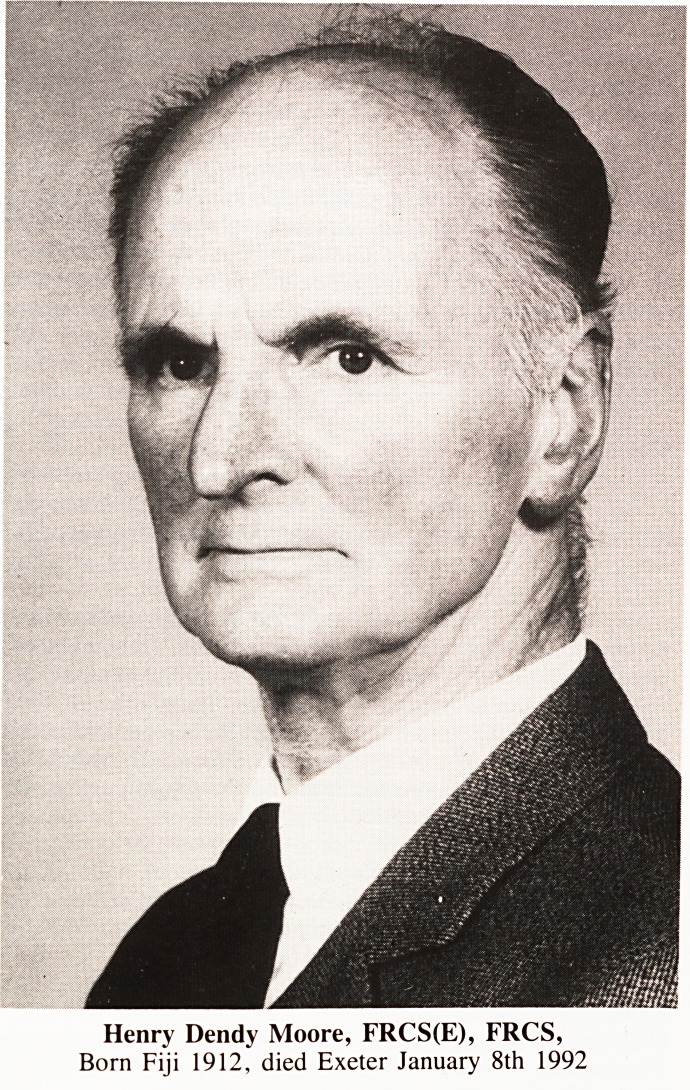# Henry Dendy Moore (1912-1992)

**Published:** 1992-03

**Authors:** 


### Obituary

**Henry Dendy Moore, FRCS(E), FRCS,**
Born Fiji 1912, died Exeter January 8th 1992

Henry was educated in Sidney NSW, qualified with a State Scholarship in 1935 and after House Appointments transferred, with two Research Fellowships, to Britain in 1938. From 1939-45 he served with RAMC in Middle East, Italy and W. Europe. Military bureaucracy exasperated him and he remained a General Duty and Regimental Medical Officer, but in Belgium collected an MC (for 'liberating a case of champagne for the Officers' Mess') and also married the sculptress Jacqueline Heyse. On demobilisation he grasped his chance, and for the next ten years worked successively as Registrar and Surgical Tutor in Professorial Units in Bristol, Edinburgh, Baltimore USA, Oxford and Leeds; so by 1955 he had had as wide and detailed experience, and published as long and varied a list of papers, as any young general surgeon in the country. He was then appointed consultant in Exeter to succeed R. Wayland Smith, at a time when District Hospitals, although settling into the vast volume of extra work presented to them in 1948, and to some extent helped by increases in medical staff, were still hampered by slow improvement in physical conditions, and new building in Exeter was nil. Moore was allotted a makeshift ward and theatre on the site of the recently bombed Exeter City Hospital (PAI) where, theatre ventilation being unsatisfactory, a meeting with SAMO took place to discuss improvements. Moore had planted under the table a smoke bomb prepared by the Works Dept., timed to go off during the meeting while the existing ventilator was working to capacity — but quite ineffectually, so after hurried evacuation the point was taken, and new equipment installed.

Moore's staff had-been warned he could be difficult, and extravagant language had been known to reduce nurses to tears; but this was soon found to be a harmless habit which ceased when things became difficult, and that throughout proceedings his anaesthetist, Rev. K. J. Powell, remained at his end relaxed, and absorbed in his Bible. Sudden demands for such bizarre items as Tampax or Eggwhisk came to be anticipated, and Moore quickly revolutionised his unit upwards to a high standard so that, with the appearance of the 'Hospital Staph.', the incidence of infection with this troublesome organism was consistently lower here than elsewhere in the area; and by 1961, to his previous repertoire which had included abdominal, urological, thyroid, vascular and Paediatric surgery, he was adding aortic grafts and removal of parathyroid tumours. In all this, a colleague describes him as 'a master of dissection, especially when tissue planes had been obliterated . . who insisted upon complete haemostasis almost as an obsession . whose **credo** was "attention to detail at all times" . . He was not afraid to flaunt convention, and early on practised conservative surgery for carcinoma of the breast, non-operative management of acute perforation of duodenal ulcer . . and he eschewed the use of drains in abdominal and biliary surgery.' A further notable contribution was the strict training he gave over 23 years, to young surgeons now themselves performing at their peak in Exeter and elsewhere in this country — and beyond. But by 1977, Parkinsonism was noticeable. New drugs were helping, and his surgical performance continued at the usual high standard; but after retirement, neurological deterioration quickened, and recent years have been painfully distressing to a crippled patient, his family and friends.

He leaves a widow, two daughters and two sons; of the latter, one is an orthopaedic surgeon, and the other a consultant in Hospicecare.

## Figures and Tables

**Figure f1:**